# Supplementary material for: Inhibition of adipocyte lipolysis by vaspin impairs thermoregulation in vivo
Source: Nat Commun. 2025 Dec 10;16:11075. doi: 10.1038/s41467-025-66950-y (PMC12698850; doi:10.1038/s41467-025-66950-y)
Supplement: Supplementary file 1 — Supplementary Infomation [file 41467_2025_66950_MOESM1_ESM.pdf]

## Supplementary Information

# Inhibition of adipocyte lipolysis by vaspin impairs thermoregulation in vivo.

Inka Rapöhn, Helen Broghammer, Anne Hoffmann, Kevin Möhlis, Anna Moormann, Isabell Kaczmarek, Doreen Thor, Henning Großkopf, Laura Krieg, Isabel Karkossa, Kristin Schubert, Martin von Bergen, Kerstin Krause, Jana Breitfeld, Peter Kovacs, Nora Klöting, Rima Nuwayhid, Stefan Langer, Adhideb Ghosh, Christian Wolfrum, Michael Stumvoll, Matthias Blüher, John T. Heiker, Juliane Weiner

## Supplementary Figures

### Supplementary Figure 1.

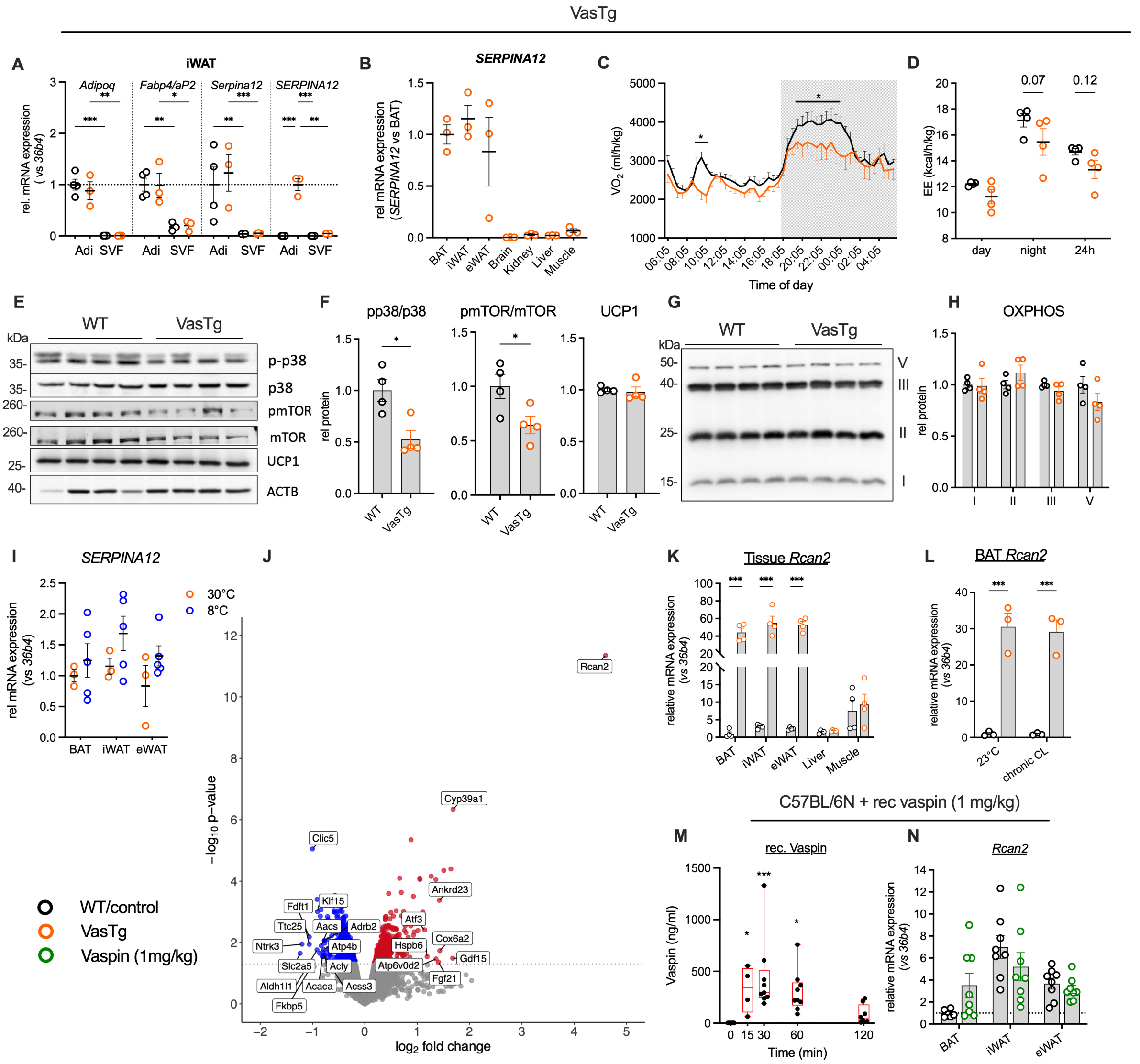


**Supplementary Figure 1.** BAT signaling and protein expression after acute cold exposure in VasTg and WT, as well as intraperitoneal vaspin-treated and control C57BL/6N mice (data from [1]). **A**) mRNA expression of adipogenic genes (*Adipoq*, *Fabp4/aP2*) and mouse and human vaspin (*Serpina12*, *SERPINA12*) mature adipocytes (Adi) and SVF in iWAT of VasTg and WT mice (n = 4/3). Gene expression is relative to WT mature adipocytes, normalized to *36b4*. **B**) mRNA

expression of human vaspin (*SERPINA12*) in different AT depots and tissues of VasTg (n = 3). Gene expression is relative to BAT, normalized to *36b4.* **C-D**) Indirect calorimetry of VasTg and WT mice under chow diet during light and dark phase (n = 4-5 per group). Oxygen consumption ($VO_2$ in ml/kg/h, over 24 h) and energy expenditure (EE in kcal/h/kg; day, night, total) was recorded over a period of 72 h. **E-F**) Western blot analysis and quantification of p-p38 MAPK, pmTOR and UCP1; (**G-H**) and OXPHOS protein expression in BAT of acutely cold-exposed (6 h) VasTg and WT mice (n = 4/4). **I**) mRNA expression of human vaspin (*SERPINA12*) in different AT depots at 30 °C and 8 °C for one week of VasTg (n = 3/3). Gene expression is relative to BAT at 30 °C, normalized to *36b4.* **J**) Volcano plot of differentially expressed genes (DEG) in BAT of acutely cold-exposed male VasTg and WT mice. Microarray gene expression data in the volcano plot are displayed as log2 fold change (FC) versus the -log10 of the p-value. Red and blue indicate up- and downregulated genes in VasTg ($p < 0.05$) compared to WT mice. **K-L**) *Rcan2* mRNA expression in BAT, eWAT, iWAT liver and muscle of VasTg and WT after acute cold exposure (n = 4/4), and in BAT of VasTg and WT mice housed at 23°C or chronically treated with CL316,243 (CL) (n = 3/3). Gene expression is relative to controls and normalized to *36b4*. **M**) Levels of human vaspin/SERPINA12 in blood of C57BL/6N mice at indicated times after vaspin injection (i.p., 1 mg/kg, n = 8); shown as box with whiskers and min/max. **N)** *Rcan2* mRNA expression in BAT, eWAT, iWAT of fasted i.p. vaspin-treated and control C57BL/6N mice after acute (6 h) cold exposure (n = 8 per group). Gene expression is relative to controls and normalized to *36b4*. Data are shown as mean ± SEM. Statistical significance was evaluated by two-way ANOVA with Tukey’s (A), or Šídák's (H-I, K, L, N) post-hoc test, or (multiple) unpaired t-tests (C-D, F) or one-way ANOVA with Holm- Šídák's post-hoc test (M). *p-value < 0.05, **p-value < 0.01, ***p-value < 0.001.

## Supplementary Figure 2.

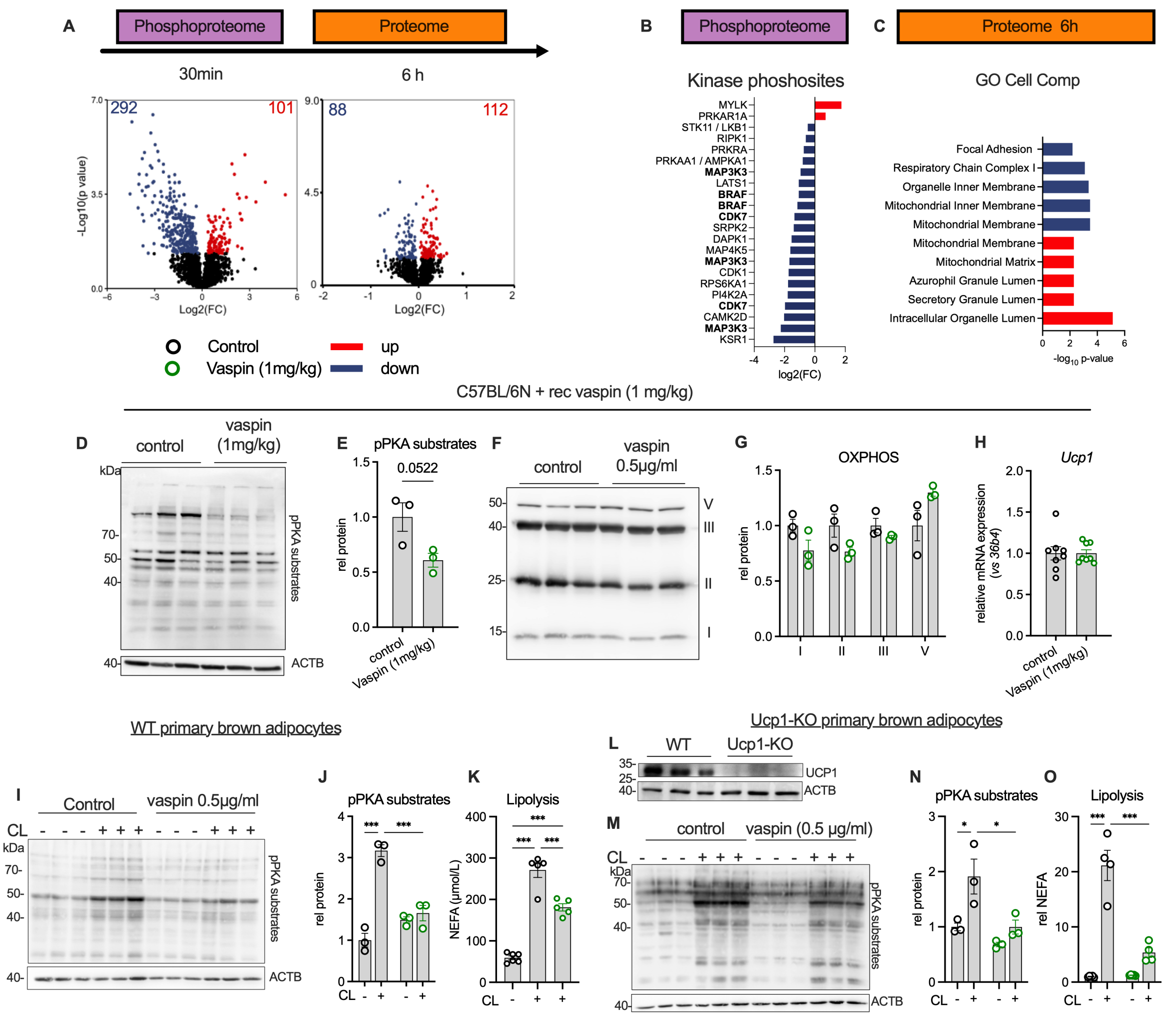


**Supplementary Figure 2. A**) Affected phosphorylation sites and proteins. Shown are significantly (FDR ≤ 0.05) altered phosphorylation sites and proteins after vaspin treatment. Log2(FCs) are depicted, highlighting the numbers of significantly increased (p-value ≤ 0.05, Log2(FC)>0) or decreased (p-value ≤ 0.05, Log2(FC)<0) sites/proteins in the corners. **B)** Kinases with significantly altered phosphorylation sites (p-value ≤ 0.05. The vertical axis states kinase specific phosphosites; the horizontal axis indicates -log10 (p-value), kinases with multiple altered phosphosites are in bold. **C)** GO term (cellular component) functional enrichment analysis of affected proteins (6 h). The vertical axis represents the GO terms significantly enriched and the horizontal axis indicates -log10 (p-value). Significantly enriched GO terms are labelled in red (increased) and blue (decreased). **D-E**) Western blot analysis (**D**) and quantification (**E**) of PKA-activation in BAT from i.p. vaspin treated WT mice after 6 h of cold exposure. **F-H)** Western blot analysis (**F**) and quantification (**G**) of OXPHOS protein expression as well as *Ucp1* mRNA Expression (**H**) in BAT from i.p. vaspin treated WT mice after 6 h of cold exposure. **I-K**) Western blot analysis (**I**) and quantification (**J**) of basal and CL-induced PKA activation, as well as free fatty acid release (NEFA, **K**, n = 5 per condition) in control and vaspin-treated differentiated primary brown adipocytes from WT mice. **L)** Western blot analysis of BAT UCP1 Expression in WT and *Ucp1*-KO mice. **M-O**) Western blot analysis (**M**) and quantification (**N**) of basal and CL-induced PKA-activation as well as free fatty acid release

(NEFA, **O**, n = 4 /4) in control and vaspin-treated differentiated primary brown adipocytes from *Ucp1*-KO mice. Data are presented as mean ± SEM of at least two (I-K) or three (M-O) independent experiments. Statistical significance was evaluated by one-way ANOVA with Tukey's (K) or two-way ANOVA with Šídák's (G) or Tukey's (J, N-O) post-hoc test, or unpaired two-tailed t-test (E, H) *p-value < 0.05, **p-value < 0.01, ***p-value < 0.001.

**Supplementary Figure 3.**

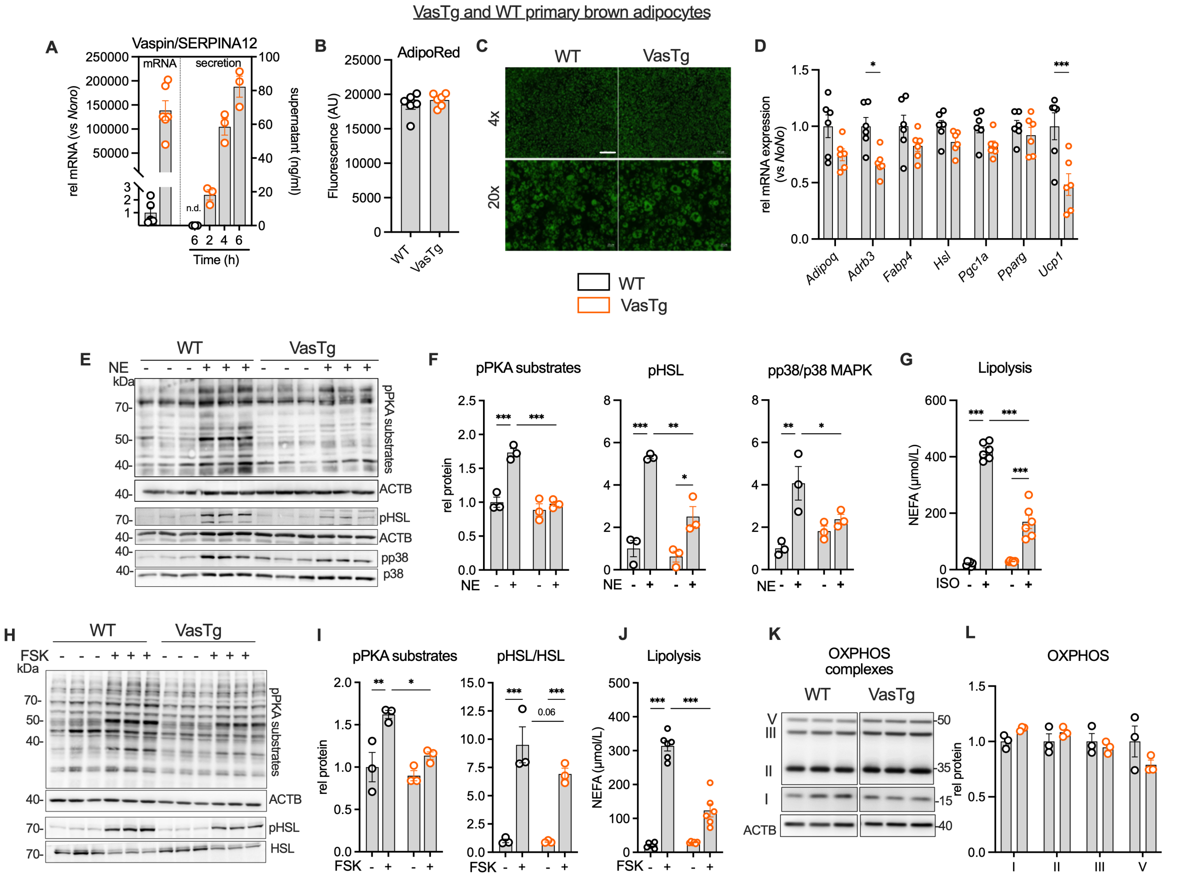


**Supplementary Figure 3.** Overexpression of vaspin suppresses adrenergic signaling and lipolysis in differentiated primary brown adipocytes. **A**) Expression and secretion of human vaspin (SERPINA12) in differentiated primary brown adipocytes from WT and VasTg mice. Gene expression is relative to WT cells and normalized to *Nono*. **B-C**) Fluorescence microscopy (**B**) and quantification (**C**) of AdipoRed-stained lipid droplets in differentiated primary brown adipocytes from VasTg and WT mice (n = 6/6). **D**) Adipogenic (*Adipoq, Fabp4, Pparg*), thermogenic (*Ucp1, Pgc1a*) and lipolytic (*Adrb3, Hsl*) gene expression in differentiated primary brown adipocytes from VasTg and WT mice (n = 6/6). Gene expression is relative to primary brown adipocytes from WT mice and normalized to *Nono*. **E-F**) Western blot analysis (**E**) and quantification (**F**) of basal and NE-induced PKA-activation (left), HSL (middle) and p38 MAPK (right) phosphorylation in differentiated primary brown adipocytes from VasTg and WT mice. **G**) Basal and ISO-induced release of free fatty acids (NEFA) in differentiated primary brown adipocytes from VasTg and WT mice (n = 6/6). **H-J**) Direct activation of ADCYs using FSK: Western blot analysis (**H**), quantification (**I-J**) of basal and FSK-induced PKA-activation (left), HSL (right) phosphorylation and free fatty acid release (NEFA, **J**, n = 6/6) in differentiated primary brown adipocytes from VasTg and WT mice. **K-L**) Western blot analysis (**K**) and

quantification (**L**) of OXPHOS proteins in differentiated primary brown adipocytes from VasTg and WT mice. Data are presented as mean ± SEM of at least two (G-I) or three (A, E, F, J) independent experiments. Statistical significance was evaluated by two-way ANOVA with Šídák's (D, L) or Tukey's (F-G, I-J) post-hoc test, or unpaired two-tailed t-test (B). *p-value < 0.05, **p-value < 0.01, ***p-value < 0.001. Scale bars: 4x: 200 µm, 20x: 50 µm.

## Supplementary Tables

**Supplementary Table 1:** Human adipose tissue sample information.

| Sample-ID | Sex | Age | BMI | Site of AT collection |
|---|---|---|---|---|
| hA1-0035 | male | 48 | 37 | Thigh |
| hA1-0036 | female | 52 | 36 | Abdomen |
| hA1-0037 | female | 44 | 36 | Back |
| hA1-0039 | female | 32 | 24 | Abdomen |

**Supplementary Table 2:** Mouse primer sequences

| Primer | Gene name | Forward 5'->3' | Reverse 3'->5' |
|---|---|---|---|
| Acly | ATP Citrat Lyase | ACCCTTTCACTGGGGATCACA | GACAGGGATCAGGTATTCCTTG |
| Adipoq | Adiponectin | AAGGACAAGGCCGTTCTCT | TATGGGTAGTTGCAGTCAGTTGG |
| Adrb3 | Beta-3 adrenergic receptor | CGCTCAACAGGTTTGATGGCT | CCAGAAGTCCTGCAAAAACGG |
| Atp1a2 | Sodium/Potassium-transport-ing ATPase subunit alpha-2 | CCACCACTGCGGAAATGG | GCCCTTAGACAGATCCACTTGG |
| Dio2 | Type II Iodothyronine Deiodi-nase | ATGGGACTCCTCAGCGTAGAC | ACTCTCCGCGAGTGGACTT |
| Fabp4 | Fatty acid binding protein 4 | AACACCGAGATTTCCTTCAA | AGTCACGCCTTTCATAACACA |
| Fasn | Fatty Acid Synthase | CTC GCT TGT CGT CTG CCT | TTG GCC CAG AAC TCC TGT AG |
| Gdp2 | Glycerol-3-Phosphate Dehydro-genase 2 | GAAGGGGACTATTCTTGTGGGT | GGATGTCAAATTCGGGTGTGT |
| Glut1 | Glucose Transporter 1 | TCAAACATGGAACCACCGCTA | AAGAGGCCGACAGAGAAGGAA |
| Glut4 | Glucose Transporter 4 | GTGGCTCTGCTGCTGCTGGAACG | GCGGGGGCCCTGGCTGAAGAG |
| Hsl | Hormone-sensitive lipase | CTGCCCAGGATTGGATGGTT | CGCTGAGGCTTTGATCTTGC |
| Pgc1a | Peroxisome proliferator-acti-vated receptor gamma coacti-vator 1-alpha | CTTTTGTGGACGGAAGCAAT | GAGTCTTGGGAAAGGACACG |
| Ppard | Peroxisome Proliferator-acti-vated Receptor delta | TCCATCGTCAACAAAGACGGG | ACTTGGGCTCAATGATGTCAC |
| Pparg | Peroxisome Proliferator-acti-vated Receptor gamma | CGTGAAGCCCATCGAGGACATC | TGGAGCAGGGGGTGAAG |
| Rcan2 | Regulator of Calcineurin 2 | CCTGCAATGTTCACCAGTCTG | TCTGTCTCTGGGGTCTGGAC |
| Ryr1 | Ryanodine Receptor 1 | CAGTTTTTGCGGACGGATGAT | CACCGGCCTCCACAGTATTG |
| Serca2b | Sarco/endoplasmic reticulum Ca2+ -ATPase | ACCTTTGCCGCTCATTTTCCAG | AGGCTGCACACACTCTTTACC |
| Ucp1 | Uncoupling Protein 1 | CCGAAACTGTACAGCGGTCT | CCGAGAGAGGCAGGTGTTTC |
| Ucp3 | Uncoupling Protein 3 | GAGATGGTGACCTACGACATCA | GCGTTCATGTATCGGGTCTTTA |

## Supplementary methods

### Untargeted proteomics

Sample preparation for proteomics was conducted as described previously [2]. Briefly, 25 µg of protein lysate from each sample (5 replicates of vaspin-treated cells and 5 replicates of media control) were reduced by adding 5 µl of 200 mM Tris(2-carboxyethyl) phosphine hydrochloride (TCEP, Sigma Aldrich, Taufkirchen, Germany) and incubated for 1 hour at 55°C. For protein alkylation, 5 µl of 375 mM iodoacetamide (Merck, Darmstadt, Germany) was added and allowed to react at room temperature for 30 minutes in darkness.

Concurrently, SpeedBeads™ magnetic carboxylated modified particles (SP3-beads, Sigma Aldrich, Taufkirchen, Germany) were prepared by washing the SP3-beads with ddH2O. Each sample was supplemented with 70 µl of acetonitrile (ACN, CAS no. 75–05-8, Merck KGaA, Darmstadt, Germany) to achieve over 50% (v/v) organic content, facilitating protein binding to the beads. After an 8-minute incubation off the magnetic rack, the supernatant was discarded following a 2-minute incubation on the magnetic rack. Two washing steps with 200 µl of 70% (v/v) ethanol (Merck KGaA, Darmstadt, Germany) in water and one washing step with 200 µl of 100% ACN ensued.

For proteolytic cleavage, 0.5 µg of trypsin (Promega GmbH, Walldorf, Germany) in 100 mM TEAB was added, maintaining a trypsin:protein ratio of 1:50, and incubated for 16 hours at 37°C.

Tandem mass tag (TMT) labeling was performed using a TMTpro-10-plex labeling reagent set (Thermo Fisher Scientific, Waltham, USA). 0.16mg of each label was added per sample, followed by a 1-hour incubation at room temperature. The labeling process was terminated by adding 1 µl of 5% hydroxylamine solution (Thermo Fisher Scientific, Waltham, USA) in 100 mM TEAB for 15 minutes. Peptide clean-up involved adding 170 µl of ACN to bind peptides to the SP3-beads (organic content >95%). After an 8-minute incubation, the samples labeled with different TMT labels were combined resulting in 5 mixtures. These mixtures were then transferred to the magnetic rack to remove the supernatant and were washed with 1 ml of ACN.

The elution of samples from the beads occurred in two fractions. Firstly, 200 µl of 87% (v/v) ACN in 10 mM ammonium formate (pH 10, Sigma Aldrich, Taufkirchen, Germany) was added to each mixture. After five rinses over the SP3-beads on the magnetic rack, the supernatant was collected as the first fraction. For the second fraction, 50 µl of ddH2O with 2% DMSO (v/v) was added to the beads of each mixture, sonicated for 1 minute, and incubated on the magnetic rack for 2 minutes. The supernatant was collected as the second fraction. Subsequently, 50 µl of ddH2O containing 2% DMSO (v/v) were added to the SP3-beads, and the supernatant was added to the second fraction. Both fractions were evaporated, dissolved in 20 µl of ddH2O, and acidified with 0.2 µl of 10% (v/v) formic acid.

All samples were analyzed on a nano ultra-performance liquid chromatography (UPLC) system (Ultimate 3000, Thermo Fischer Scientific) coupled on-line to an Orbitrap mass spectrometer (Thermo Fischer Scientific) equipped with a chip-based ESI source (TriVersa NanoMate, Advion,). Peptides (10 µg) were trapped on a C18 column (Acclaim PepMap 100 C18, nanoViper, 2 µm, 75 µm × 5 cm, Thermo Fisher Scientific, #164535) and separated on a

reversed-phase C18 column (Acclaim PepMap 100 C18, nanoViper, 3 µm, 75 µm × 25 cm, Thermo Fisher Scientific, #164569). A non-linear gradient of 160 min and a flow rate of 0.3 µl/min was used for separation of the peptides. Mass spectra were acquired in a data-dependent manner. For MS1 scans, the m/z ratio was set to 350–1550 m/z, resolution to 120 K, automatic gain control (AGC) to 3× 106 ions and maximum injection time to 120 ms. The top 15 most abundant ions were selected for high-energy collisional dissociation, using a window of 0.7 m/z, NCE 34, resolution 60 K, AGC target 2× 105 ions and maximum injection time 120 ms. The dynamic exclusion was set to 45 s.
Samples were analyzed using MS parameters described before [2]. MS raw data were processed using Proteome-Discoverer 2.4.0.350. The database search was conducted against the UniprotKB/Swissprot reference proteome of Mus muculus (19th of November 2019). The resulting replicate-wise TMT-reporter ion intensity FCs of each timepoint (vaspin vs control) were used for subsequent analyses.
Statistical analysis was carried out in R-3.6.1 according to the workflow described in the package proteomicsr [3]. Data were Log2-transformed, filtered for proteins quantified in at least 3 of the 5 replicates, and variance-stabilized. This resulted in FCs for 2327 proteins. Significant changes were calculated using Student's t-test (p value ≤ 0.05). For pathway enrichment analysis the Enricher tool was used with the definition of "mouse" as organism using significantly altered proteins. Benjamini & Hochberg adjusted p-values for up and downregulated pathways were extracted and used for visualizations in GraphPadPrism 10.

**Phosphoproteomics**

For analysis of the phosphoproteome, samples were prepared as described previously [4]. Briefly, 564µg protein from each sample was precipitated using SpeedBeads™ magnetic carboxylated modified particles (SP3-beads, Sigma Aldrich, Taufkirchen, Germany), then subjected to reduction with 50 mM TCEP (Sigma-Aldrich, USA), followed by carbamidomethylation with 100 mM IAA (Merck, Germany), and enzymatic digestion with Trypsin (Promega, USA) at a 1:50 Trypsin/protein ratio. The resulting peptides were collected and completely dried using a vacuum concentrator.
Phosphopeptides were enriched in two sequential steps using TiO2- and Fe-NTA-based affinity chromatography. Initially, the High-Select™ TiO2 Phosphopeptide Enrichment Kit (Thermo Scientific, USA) was employed as per the manufacturer's instructions. The flow-through from this step, along with the first wash fractions, was pooled and dried for the subsequent enrichment. Subsequently, the High-Select™ Fe-NTA Phosphopeptide Enrichment Kit (Thermo Scientific, USA) was utilized according to the manufacturer's guidelines. The enriched phosphopeptide eluates from both steps were combined, dried, and reconstituted in 0.1 % FA for LC-MS/MS analysis. Phosphopeptide enrichment was conducted in 5 replicates each of vaspin-treated cells and media controls.

All samples were analysed on the same instrument as the proteome samples. A tripartite linear 145 min gradient starting from 4 % eluent B (0.1 % FA in 80 % ACN) in eluent A (0.1 % FA in water) to 55 % eluent B via 18 % eluent B after 77.5 min and 30 % eluent B after 115 min was used. After each sample, the column was flushed to 99% eluent B and reconstituted to

starting conditions. Mass spectra were acquired in a data-dependent manner. For MS1 scans the following parameters were set: m/z range 350-1550, maximum injection time = 120 ms, AGC = 3x106, R = 120 000. The top 15 most abundant ions were selected for MS2 acquisition using the following parameters: isolation window of 0.7 m/z, maximum injection time 150 ms, AGC = 2x105, normalized collision energy (NCE) = 28, R = 15 000, m/z range 200-2000. Fragmented ions were dynamically excluded for 45 s.
LC-MS/MS raw data underwent analysis using MaxQuant (Version 1.6.7.0). The database search was conducted against the UniprotKB/Swissprot reference proteome of Mus muculus (17th of February 2020). Search parameters included maximum missed cleavages = 2, minimal peptide length = 6 amino acids, and various tolerances for peptide and fragment mass spectrometry. Filtering and quantification were performed using label-free quantification (LFQ) and stringent criteria for reliable quantification.

Further statistical analysis was conducted using R-3.6.1 with the same packages as used in the proteome analysis. Proteins and phosphosites identified by site, from the reverse database, or as potential contaminants were excluded. Significantly altered proteins and phosphosites were identified using Student's t-test (p value ≤ 0.05). Kinase activities were inferred by integrating amino acid sequence windows of significantly altered PP-sites with kinase-substrate motifs using KinSwingR [5] in the R environment, based on the kinase-substrate dataset from PhosphoSitePlus. For pathway enrichment analysis the Enricher tool was used according to the proteome analysis.

**References**

1. Rapohn, I., et al., *Overexpressing high levels of human vaspin limits high fat diet-induced obesity and enhances energy expenditure in a transgenic mouse.* Front Endocrinol (Lausanne), 2023. **14**: p. 1146454.
2. Raps, S., et al., *Triclosan and its alternatives, especially chlorhexidine, modulate macrophage immune response with distinct modes of action.* Sci Total Environ, 2024. **914**: p. 169650.
3. Karkossa, I., *proteomicsr: An analysis pipeline for label-based and label-free proteomics data (1.0.0).* Zenodo, 2023( doi.org/10.5281/zenodo.10171433).
4. Grosskopf, H., et al., *Non-Genomic AhR-Signaling Modulates the Immune Response in Endotoxin-Activated Macrophages After Activation by the Environmental Stressor BaP.* Front Immunol, 2021. **12**: p. 620270.
5. Waardenberg, A., *KinSwingR: network-based kinase activity prediction.* R package version 1.22.0., 2024.
