# Supplementary material for: Inhibition of adipocyte lipolysis by vaspin impairs thermoregulation in vivo
Source: Nat Commun. 2025 Dec 10;16:11075. doi: 10.1038/s41467-025-66950-y (PMC12698850; doi:10.1038/s41467-025-66950-y)
Supplement: Supplementary file 2 — Description of Additional Supplementary Files [file 41467_2025_66950_MOESM2_ESM.pdf]

Supplementary Data Legends:

Supplementary Data 1: Differentially expressed genes in BAT of VasTg and WT mice after cold exposure ($p<0.05$).

Supplementary Data 2: Pathway analysis of differentially expressed genes in BAT of VasTg and WT mice after cold exposure using the KEGG_2019_mouse database.

Supplementary Data 3: Phosphoproteomics, differentially abundant phosphorylation sites in control and 30 min vaspin-treated imBA with $p<0.05$.

Supplementary Data 4: Pathway analysis of differentially phosphorylated proteins in imBA after 30min vaspin treatment using the KEGG_2019_mouse database.

Supplementary Data 5: Kinases with differentially phosphorylated sites ($p<0.05$) in imBA after 30min vaspin treatment.

Supplementary Data 6: Proteomics, differentially abundant proteins in control and 6 h vaspin-treated imBA with padj.<0.05.

Supplementary Data 7: Pathway analysis of proteins with significantly altered abundance in imBA after 6h vaspin treatment using the KEGG_2019_mouse database.
