# Supplementary material for: Inhibition of adipocyte lipolysis by vaspin impairs thermoregulation in vivo
Source: Nat Commun. 2025 Dec 10;16:11075. doi: 10.1038/s41467-025-66950-y (PMC12698850; doi:10.1038/s41467-025-66950-y)
Supplement: Supplementary file 4 — Reporting Summary [file 41467_2025_66950_MOESM4_ESM.pdf]

nature portfolio

Corresponding author(s): John Heiker, Juliane Weiner

Last updated by author(s): Sep 8, 2025

Reporting Summary

Nature Portfolio wishes to improve the reproducibility of the work that we publish. This form provides structure for consistency and transparency in reporting. For further information on Nature Portfolio policies, see our Editorial Policies and the Editorial Policy Checklist.

Statistics

For all statistical analyses, confirm that the following items are present in the figure legend, table legend, main text, or Methods section.

n/a	Confirmed	
☐	☒	The exact sample size (*n*) for each experimental group/condition, given as a discrete number and unit of measurement
☐	☒	A statement on whether measurements were taken from distinct samples or whether the same sample was measured repeatedly
☐	☒	The statistical test(s) used AND whether they are one- or two-sided<br>*Only common tests should be described solely by name; describe more complex techniques in the Methods section.*
☒	☐	A description of all covariates tested
☒	☐	A description of any assumptions or corrections, such as tests of normality and adjustment for multiple comparisons
☐	☒	A full description of the statistical parameters including central tendency (e.g. means) or other basic estimates (e.g. regression coefficient) AND variation (e.g. standard deviation) or associated estimates of uncertainty (e.g. confidence intervals)
☐	☒	For null hypothesis testing, the test statistic (e.g. *F*, *t*, *r*) with confidence intervals, effect sizes, degrees of freedom and *P* value noted<br>*Give P values as exact values whenever suitable.*
☒	☐	For Bayesian analysis, information on the choice of priors and Markov chain Monte Carlo settings
☒	☐	For hierarchical and complex designs, identification of the appropriate level for tests and full reporting of outcomes
☒	☐	Estimates of effect sizes (e.g. Cohen's *d*, Pearson's *r*), indicating how they were calculated

Our web collection on statistics for biologists contains articles on many of the points above.

Software and code

Policy information about availability of computer code

Data collection	Seahorse: Wave Software Pro (Version 10.1.0.1); Western Blot: Genetools from Syngene (File version 4.03.05.0) ; PCR: LightCycler 480 Software Version 1.5.1.62;Thermal imaging: InfraTec IBRIS3.1 Professional; Absorbance, Fluorescence measurements: SoftMax Pro GxP Software 7.
Data analysis	Data analysis was performed using GraphPad Prism version 10.6.0. Data analysis for proteomic and trasncriptomic data is described in detail in the manuscript.

For manuscripts utilizing custom algorithms or software that are central to the research but not yet described in published literature, software must be made available to editors and reviewers. We strongly encourage code deposition in a community repository (e.g. GitHub). See the Nature Portfolio guidelines for submitting code & software for further information.

Data

Policy information about availability of data

All manuscripts must include a data availability statement. This statement should provide the following information, where applicable:

- Accession codes, unique identifiers, or web links for publicly available datasets
- A description of any restrictions on data availability
- For clinical datasets or third party data, please ensure that the statement adheres to our policy

There are no restrictions as to the availability of materials reported in the manuscript. The data that support the findings of this study are available from the

corresponding author on request.
Microarray data have been deposited in the ArrayExpress database at EMBL-EBI (www.ebi.ac.uk/arrayexpress) under accession number E-MTAB-14068.
The mass spectrometry proteomics data have been deposited to the ProteomeXchange Consortium (http://proteomecentral.proteomexchange.org) via the PRIDE partner repository with the dataset identifiers PXD068908 and PXD1-20250930-090155-2198754

Research involving human participants, their data, or biological material

Policy information about studies with human participants or human data. See also policy information about sex, gender (identity/presentation), and sexual orientation and race, ethnicity and racism.

Reporting on sex and gender	SAT samples were randomly collected and were from 3 female and one male patient. Sex was reported based on self-reporting.
Reporting on race, ethnicity, or other socially relevant groupings	This information was not collected.
Population characteristics	Patients were between 32 and 52 years old, with BMI ranging from 23.9 to 36.6. All information is given in Supplementary table 8.
Recruitment	Samples were collected during elective aesthetic and post-bariatric surgery at the Division of Plastic, Aesthetic and Special Hand Surgery of University Hospital Leipzig between January and April 2024.
Ethics oversight	Leipzig Obesity BioBank (LOBB): Written informed consent was obtained from all patients. All studies were approved by the Ethics Committee of the University of Leipzig (approval numbers: 159-12-21052012 and 017-12ek) and performed in accordance with the Declaration of Helsinki, the Bioethics Convention (Oviedo), and EU Directive on Clinical Trials (Directive 2001/20/EC). All AT donors have been informed of the purpose, risks and benefits of the biobank. Ethical guidelines and EU legislation for privacy and confidentiality in personal data collection and processing is being followed, in particular directive 95/46/EC.

Note that full information on the approval of the study protocol must also be provided in the manuscript.

Field-specific reporting

Please select the one below that is the best fit for your research. If you are not sure, read the appropriate sections before making your selection.

☒ Life sciences ☐ Behavioural & social sciences ☐ Ecological, evolutionary & environmental sciences

For a reference copy of the document with all sections, see nature.com/documents/nr-reporting-summary-flat.pdf

Life sciences study design

All studies must disclose on these points even when the disclosure is negative.

Sample size	This study did not use statistical methods to determine the experimental sample size in advance, but referred to previous related studies. The sample sizes used in each set of experiments in this study are displayed in the figures and legends.
Data exclusions	Given a high variability in the expression levels of genes and proteins, some data from gene expression analyses had to be excluded based on outlier identification (GraphPad Prism). To assure reproducibility and robustness of the results, these analyses were repeated. Only healthy mice were used in the study.
Replication	All data reported in this study were reproduced as biological replicates as stated in the methods. All in vitro experiments were replicated at least twice except for -omics analyses and where stated otherwise.
Randomization	For in vivo studies, mice were age-, sex-, and genotype-matched. Samples derived from tissue collection were processed in random order.
Blinding	Data collection and analysis were not performed blind to the conditions of the experiments

Reporting for specific materials, systems and methods

We require information from authors about some types of materials, experimental systems and methods used in many studies. Here, indicate whether each material, system or method listed is relevant to your study. If you are not sure if a list item applies to your research, read the appropriate section before selecting a response.

Materials & experimental systems

n/a	Involved in the study
☐	☒ Antibodies
☐	☒ Eukaryotic cell lines
☒	☐ Palaeontology and archaeology
☐	☒ Animals and other organisms
☐	☒ Clinical data
☒	☐ Dual use research of concern
☒	☐ Plants

Methods

n/a	Involved in the study
☒	☐ ChIP-seq
☒	☐ Flow cytometry
☒	☐ MRI-based neuroimaging

Antibodies

Antibodies used	The following primary antibodies were used: from Cell Signaling Technologies: phospho-PKA substrates (RRXS*/T*, #9624), HSL (#4107), phospho-HSL (Ser660, #4126), p38 MAPK (#9212), phospho-p38 MAPK (Thr180/Tyr182, #4511), anti-rabbit-HRP (#7074), anti-mouse-HRP (#7076); from Abcam: UCP1 (ab10983); from Sigma-Aldrich, St. Louis, MO, USA: ACTB (#A1978); from Thermo Fisher Scientific: OXPHOS (#45-8099). Human vaspin in serum or cell supernatants were measured by ELISA (human vaspin, Adipogen).
Validation	Validation of commercial antibodies was based on information provided by the manufacturer and/or previous use in our laboratory (e.g. knock out validated, overepression validated).

Eukaryotic cell lines

Policy information about cell lines and Sex and Gender in Research

Cell line source(s)	Primary brown adipocytes from male and female C57BL/6N mice, VasTg mice or WT littermates, as well as immortalized brown adipocytes (imBA, originating from C. Ronald Kahns lab (Klein et al. 2002) were used. Human adipocytes were isolated from subcutaneous adipose tissue. Cells were cultured as explained in detail in the manuscript.
Authentication	The cell lines used during this research were not authenticated in our lab, but expression of cell-specific human/mouse gene marker genes was assessed as per routine during our research.
Mycoplasma contamination	All experiments were performed with mycoplasma-free cells, regularly tested using the Mycoalert Kit (Lonza, Basel, Switzerland).
Commonly misidentified lines (See ICLAC register)	No misidentified cell lines were used during this research.

Animals and other research organisms

Policy information about studies involving animals; ARRIVE guidelines recommended for reporting animal research, and Sex and Gender in Research

Laboratory animals	C57BL/6NTac mice (Taconic Bioscience, Lille Skensved, Denmark) and adipose-tissue specific human vaspin-transgenic mice on the C57BL/6N background (VasTg, described in Rapöhn et al 2023) were bred at the Sächsische Inkubator für Klinische Translation (SIKT), Leipzig.
Wild animals	The study did not involve wild animals.
Reporting on sex	Although the mice used in in vivo studies were female mice, our research findings are not limited to a specific gender,. sSex was not a factor in our study design involving vertebrates, primary cell lines, and human participants.
Field-collected samples	The study did not involve field-collected samples.
Ethics oversight	Animal studies: All animal experiments were approved by the local authorities of the Free State of Saxony, Germany (Landesdirektion Leipzig: TVV39/14; TVV26/16, T09/21), as recommended by the responsible local animal ethics review board.

Note that full information on the approval of the study protocol must also be provided in the manuscript.

Clinical data

Policy information about clinical studies

All manuscripts should comply with the ICMJE guidelines for publication of clinical research and a completed CONSORT checklist must be included with all submissions.

Clinical trial registration	n.a.
Study protocol	*Note where the full trial protocol can be accessed OR if not available, explain why.*

Data collection	*Describe the settings and locales of data collection, noting the time periods of recruitment and data collection.*
Outcomes	*Describe how you pre-defined primary and secondary outcome measures and how you assessed these measures.*

Plants

Seed stocks	n.a.
Novel plant genotypes	n.a.
Authentication	n.a.
